# Supplementary material for: Chain-End Effects on Supramolecular Poly(ethylene glycol) Polymers
Source: Polymers (Basel). 2021 Jul 7;13(14):2235. doi: 10.3390/polym13142235 (PMC8309292; doi:10.3390/polym13142235)
Supplement: Supplementary file 1 [file polymers-13-02235-s001.zip › polymers-1276978-supplementary.pdf]

Supporting Information for

Chain-End Effects on Supramolecular Poly (Ethylene Glycol) Polymers

Ana Brás [1,*], Ana Arizaga [1], Uxue Agirre [1], Marie Dorau [1], Judith Houston [2], Aurel Radulescu [2], Margarita Kruteva [3], Wim Pyckhout-Hintzen [3] and Annette M. Schmidt [1]

[1] Institute of Physical Chemistry, University of Cologne, 50939 Cologne, Germany; ana.arizaga@gmail.com (A.A.); uagirre@smail.uni-koeln.de (U.A.); MarieDorau@gmx.net (M.D.); annette.schmidt@uni-koeln.de (A.M.S.)

[2] Jülich Centre for Neutron Science (JCNS-1) at Heinz Maier Leibnitz-Zentrum (MLZ), Forschungszentrum Jülich GmbH, 85748 Garching, Germany; judith.houston@esss.se (J.H.); a.radulescu@fz-juelich.de (A.R.)

[3] Jülich Centre for Neutron Science (JCNS-1), Forschungszentrum Jülich GmbH, 52428 Jülich, Germany; m.kruteva@fz-juelich.de (M.K.); w.pyckhout@fz-juelich.de (W.P.-H.)

* Correspondence: ana.eliasbras@uni-koeln.de; Tel.: +49-221-470-5473

I. SUPRAMOLECULAR POLYMER SYNTHESIS AND CHARACTERIZATION

Materials

2-amino-4-hydroxy-6-methylpyrimidine (Acros organics®, 99%), Ammonium hydroxide solution (Sigma-Aldrich®, 28%-30%), 2-chloro-4,6-diamino-1,3,5-triazine (Sigma-Aldrich®, 95%), Chloroform anhydrous (Sigma-Aldrich®, ≥ 99%), Chloroform HPLC (Fisher Scientific®, 99,8%), D-chloroform (Deutero GmbH, 99,8%), Dibutyltin dilaurate (Sigma-Aldrich®, 95%), Dichloromethane anhydrous (Sigma-Aldrich®, 99,8%), Dichloromethane HPLC (Fisher Scientific®, 99,99%), Dimethyl formamide anhydrous (Sigma-Aldrich®, 99,8%), Dimethyl sulfoxide-D6 (Deutero GmbH, 99,5%), Ethanol (Fisher Scientific®, 99,99%), 1,6-hexane diisocyanate (Sigma-Aldrich®, 99%), Methanesulfonyl chloride (Sigma-Aldrich®, 99,7%), N,N-diisopropylethylamine (Sigma-Aldrich®, ≥ 99%), O-(Benzotriazol-1-yl)-N,N,N′,N′-tetramethyluronium tetrafluoroborate (Sigma-Aldrich®, ≥ 97%), Pentane HPLC (Fisher Scientific®, > 99%), Poly(ethylene glycol) (Merck®),Silica gel (Acros organics®), Sodium hydrogencarbonate (Sigma-Aldrich®), Sodium sulfate (Sigma-Aldrich®), Tetrahydrofuran (BDH Prolabo®, 99,9%), Thymine-1-acetic acid (Sigma-Aldrich®, 98%), Triethylamine (Sigma-Aldrich®, 99%).

Drying and purification procedures

Most of the chemicals above are used as received, except a few of them, which need to be pre-dried. Polyethylene glycol 2.000 (Merck®) is dried in vacuum for two days. N,N-diisopropylethylamine, methanesulfonyl chloride and triethylamine are pre-dried while bubbling argon for a few minutes under inert atmosphere. 2-amino-4-hydroxy-6-methylpyrimidine, O-(benzotriazol-1-yl)-N,N,N′,N′-tetramethyluronium tetrafluoroborate and thymine-1-acetic acid are pre-dried in vacuum for ca. 30 minutes. Tetrahydrofuran is heated in reflux with sodium and finally distilled.

Synthesis of H_2N-PEG-NH_2

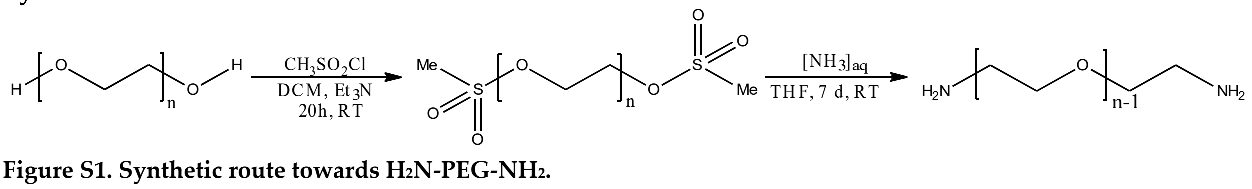

Figure S1. Synthetic route towards H_2N-PEG-NH_2.

This synthesis is performed following the instructions described by Kruteva et al [1]. 15 g (8.23 mmol, 1 eq.) of pre-dried Polyethylene glycol are placed in a two-neck one-Schlenk flask and dissolved in 225 mL dichloromethane. Then 15.75 mL (113.62 mmol, 13.8 eq.) of triethylamine are added and afterwards 4.65 mL (60.08 mmol, 7.3 eq.) of methansulfonyl chloride are added dropwise at 0°C under vigorous stirring. After 20 h of stirring at room temperature the solvent is removed by evaporation and the intermediate product, an orange solid, is obtained.

The solid residue from previous step is dissolved in 200 mL of pre-dried tetrahydrofuran, and dropwise added to 800 mL (6164.38 mmol, 749 eq.) of ammonium hydroxide solution (30%). The mixture is stirred at room temperature for 7 days. Subsequently the polymer is extracted three times with dichloromethane, and the combined organic phases are

washed with water. The solution is dried over magnesium sulfate, the solvent is removed under reduced pressure and the product is obtained as a white solid. The functionalization degree with amino groups was calculated from the ^{1}H-NMR spectrum in DMSO-d_6 by comparing the signals of the methylene groups next to the NH_2–end groups at 2.69 ppm and next to the residual OH–end groups at 3.51 ppm. The functionalization degree with amino groups was found to be 93%.

Synthesis of Thy-PEG-Thy

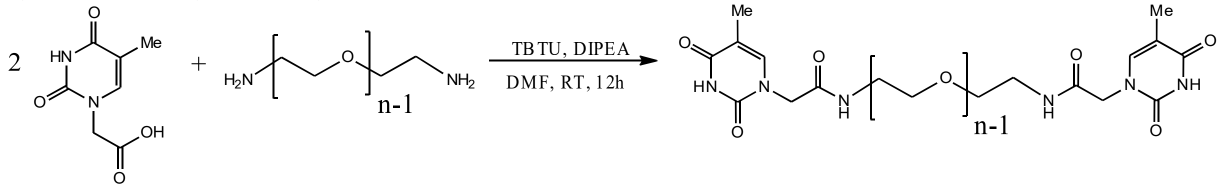

Figure S2. Synthetic route towards Thy-PEG-Thy.

The synthesis of PEG-Thy is done by performing the procedure described by Kruteva et al [1]. 10 g of polyethylene glycol diamine (5.49 mmol, 1 eq), 1.935 g of thymine-1-acetic acid (10.5 mmol, 1.9 eq), 3.85 g (11.99 mmol, 2.2 eq) of TBTU and 3.875 g (30 mmol, 5.5 eq) of N,N-diisopropylethylamin are placed in a flask and three cycles vacuum/Ar were preformed. Then, the reagents are dissolved in 50 mL of DMF and the mixture is stirred at room temperature overnight. Afterwards, 50 mL of water are added and the solvent is removed under reduced pressure. The residue is dissolved in dichloromethane, washed with water and dried over sodium sulfate. The solvent is removed under reduced pressure and the raw product is recrystallized from ethanol at -28 °C. After drying the product, a light yellow solid, is obtained. In the DMSO-d_6 ^{1}H-NMR spectrum the thymine protons are assigned to the following chemical shifts: 8.21 ppm (NH-CO-), 7.42 ppm (=CH), 4.27 ppm (CO-CH_2-N), 1.75 ppm (CH_3). The functionalization degree with amino groups was found to be 90%.

Synthesis of dat-PEG-dat

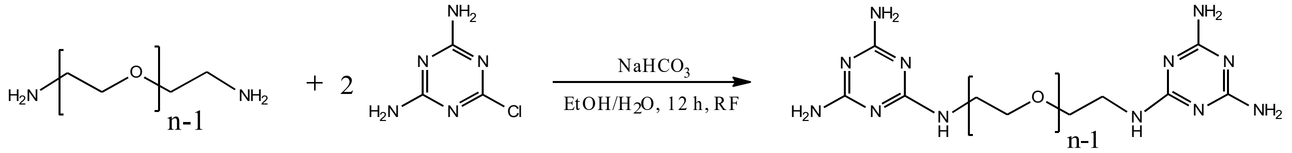

Figure S3. Synthetic route towards dat-PEG-dat.

Kruteva et al [1] describes the synthesis of PEG-dat as follows: 10 g of polyethylene glycol diamine (5.49 mmol, 1 eq) are dissolved in a mixture of 37.5 mL of ethanol and 37.5 mL of water. Then 1.6 g (11 mmol, 2 eq) of 2-chloro-4,6-diamino-1,3,5-triazine and 0.185 g (11 mmol, 2 eq) of sodium hydrogen carbonate are added and the mixture was headed to reflux under magnetic stirring overnight. Afterwards, the solvent and the volatile materials are removed under reduced pressure, and the residue is dissolved in dichloromethane. The solution is washed with water, dried over sodium sulfate, and the solvent is removed under reduced pressure. Finally the product, a colourless solid, is received. In the DMSO-d_6 ^{1}H-NMR spectrum the DAT protons are assigned to the following chemical shifts: 6.31 ppm (-CH_2NH-), 6.10 and 5.95 ppm (-NH_2), 3.51 ppm (terminal -CH_2 in PEG). The functionalization degree with amino groups was found to be 93%.

Synthesis of 2(6-isocyanathexylaminocarbonylamin)-6-methyl-4-pyrimidone

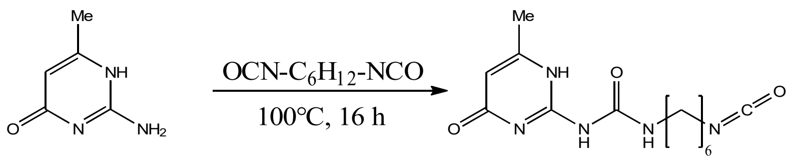

Figure S4. Synthetic route towards 2(6-isocyanathexylaminocarbonylamin)-6-methyl-4-pyrimidone.

The synthesis of 2(6-isocyanathexylaminocarbonylamin)-6-methyl-4-pyrimidone is performed following the procedure described in [2]. In a two- neck one- Schlenk flask, 2.5 g (20 mmol, 1 eq) of 2-amino-4-hydroxy-6-methylpyrimidine are dissolved in 22 mL (137 mmol, 6.85 eq) of 1,6-hexane diisocyanate. The mixture is headed up to 100 °C and was stirred at this temperature for 16 h. After cooling down, pentane is added and the mixture is filtered by use of a glass filter frit. The polymer is washed several times with pentane, and afterwards the product is dried 48h in a vacuum oven at 50 °C. In the $CDCl_3$ ^{1}H-NMR spectrum the protons were assigned to the following chemical shifts: 13.13 (-CH_3C**NH**-), 11.88 (-CH_2NH(C=O)**NH**-), 10.28 (- CH_2**NH**(C=O)NH-), 5.84 (-(C=O)**CH**=CCH_3), 3.33 (- NH(C=O)NH**CH_2**-), 2.25 (-**CH_3**C=CH-), 1.66 – 1.61 (-NCH_2**CH_2**-), 1.43 (-CH_2CH_2**CH_2CH_2**CH_2CH_2-).

Synthesis of upy-PEG-upy

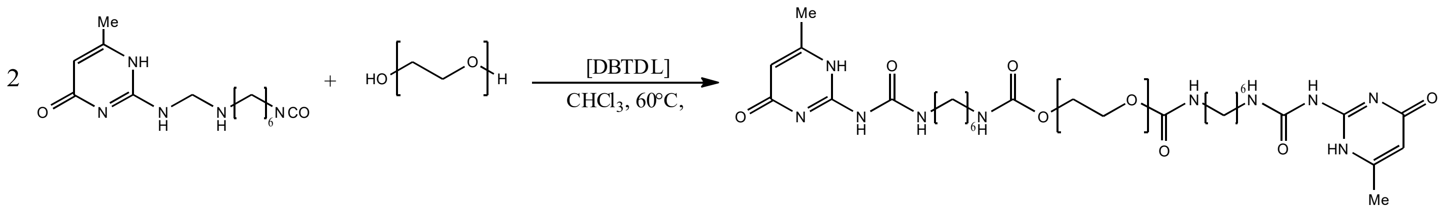

Figure S3. Synthetic route towards upy-PEG-upy.

1.7 g (0.85 mmol; 1 eq.) polyethylene glycol 2000 (Merck®) were dissolved in 20 mL (c = 42.5 mol/L) of dry $CHCl_3$ and 1 g (3.41 mmol; 4 eq.) 2(6-isocyanathexylaminocarbonylamin)-6-methyl-4[1H]pyrimidinone dissolved in 8 mL dry $CHCl_3$ were added. To prepare the catalyst 2 drops Dibutyltin dilaurate were dissolved in 0.35 mL of $CHCl_3$ and 2 drops of the catalyst solution were added to the reaction mixture. After 16 h stirring at 60°C, the cold mixture was diluted with 28 mL $CHCl_3$, filtered and washed. Afterwards, the sample was evaporated to a total volume of 28 mL and 0.284 g silica gel and 1 drop catalyst were inserted. This solution was stirred at 60°C for 1 h and, when cold, filtered and washed. Finally the solvent was removed under reduced pressure and the product was dried 48 h in vacuum at 50°C. In the $CDCl_3$ ^{1}H-NMR spectrum the upy protons were assigned to the following chemical shifts: δ = 13.13 (-CH3C**NH**-), 11.87 (-CH_2NH(C=O)**NH**-), 10.15 (- CH_2**NH**(C=O)NH-), 5.04 (**NH**(C=O)O), 4.22 (-**CH_2**O(C=O)NH-), 3.27 + 3.25 (-NH(C=O)NH**CH_2** + **CH_2**NH(C=O)O-), 2.25 (-**CH_3**C=CH-), 1,18 (- NCH_2**CH_2**-).The functionalization degree with amino groups was found to be 99%.

II. Theoretical Approach

Random Phase Approximation (RPA) Model for multicomponent systems

The observation of a block copolymer-like scattering signal on supramolecular polymers requires the use of random phase approximation (RPA) formalism for multicomponent systems as has already been shown in literature [1,3,4]. In this work we consider the binary system of a multiblock copolymer, as A block being the PEG polymer and B block representing both associating end-groups into a single effective block, basing on the identical solubility parameters. Thereby the system can be modelled as the general multiblock copolymer as $(AB)_X$ copolymer with association degree X of diblock units, representing the number of aggregated building blocks N_{agg}. We have thus applied a full two-component RPA including all interactions χ_{ij} with i, j = A, B. With the number of monomers denoted as N_A and N_B (polymer and compound end- groups, respectively), the volume fractions ϕ_A, and ϕ_B, specific monomeric volumes v_A, and v_B, form

factors $P_{AA}(Q)$, $P_{BB}(Q)$ and $P_{AB}(Q)$, the interaction parameters χ_{AB} polymer–end groups, the contrast $\Delta\rho^2 = (\rho_A - \rho_B)^2$ and the number of aggregates N_{agg}, the structure factor is now given as:

$$I(Q)/\Delta\boldsymbol{\rho}^2 = S_{RPA} = \frac{S^0_{AA}S^0_{BB}-S^{0^2}_{AB}}{(S^0_{AA}+S^0_{BB}+2S^0_{AB})-2\frac{\chi_{AB}}{\sqrt{v_A v_B}}\left(S^0_{AA}S^0_{BB}+S^{0^2}_{AB}\right)} \quad (1)$$

The ideal noninteracting structure factors for an incompressible binary blend in the homogeneous phase are given *by:*

$$S^0_{AA} = N_A\phi_A v_A P_{AA} \quad (2)$$

$$S^0_{BB} = N_B\phi_B v_B P_{BB} \quad (3)$$

$$S^0_{AA} = \sqrt{N_A N_B \phi_A \phi_B v_A v_B} P_{AB} \quad (4)$$

which are inserted in the RPA expression above (Eq. (1)). Using the dimensionless parameter $a = (Q^2 l^2_{st})/6$ such that $R^2_g = aN$, where N is the specified "block length" (equivalent to the number of repeating monomer units of the polymer, in the case of PEG or N_A and to three repeating PEG monomer units in the case of N_B) and reflecting the random-walk statistics of the building blocks, with l_{st} the effective statistical segment length of the Gaussian subchains resulting from the approximation of including the end groups into the more flexible building blocks, we obtain:

$$P_{AA} = N_{agg}G(N_A) + \sum_{i=1}^{N_{agg}-1}\left(2N_{agg} - 2i\right) F^2(N_A)E^i(N_B) \times E^{(i-1)}(N_A) \quad (5)$$

$$P_{BB} = \left(N_{agg} - 1\right)G(N_B)\sum_{i=1}^{N_{agg}-2}\left(2\left(N_{agg} - 1\right) - 2i\right) F^2(N_B)E^i(N_A) \times E^{(i-1)}(N_B) \quad (6)$$

$$P_{AB} = \sum_{i=1}^{N_{agg}-1}\left(2N_{agg} - 2i\right) F(N_A)F(N_B)(E(N_B \times E(N_B))^{(i-1)} \quad (7)$$

With

$$G(a,N) = \frac{2(\exp(-aN)+aN-1)}{(aN)^2} \quad (8)$$

$$F(a,N) = \frac{1-\exp(-aN)}{(aN)} \quad (9)$$

$$E(a,N) = \exp(-aN) \quad (10)$$

The individual blocks are taken as monodisperse. More details on the RPA model can be found in [1] and [3].

Percus-Yevick (PY) Approximation Model

The model assumes a suspension of hard spheres with repulsive interaction. The potential is infinite when two spheres touch each other. The scattering intensity per unit of volume of a spherically symmetric particles as in the Percus-Yevick hard sphere model can be written as [5]:

$$I(Q) = \phi\Delta\rho^2 V F(Q,R_c)^2 S(Q,R_d) \quad (11)$$

where ϕ is the volume fraction of particles, $\Delta\rho^2$ the contrast factor between polymer and upy groups forming the spherical particles and V is the molecular volume of the particle. $P(Q,R_c) = F(Q,R_c)^2$ is the form factor and $S(Q,R_d)$ the structure factor due to the contribution of the interactions between particles. Basically, the scattering intensity of the disordered particles can be attributed to the form factor of spheres with radius R_c (the spherical clusters) and the structure factor of hard spheres (the Percus-Yevick correlation radius due to the interactions between spherical clusters) with a radius of R_c. At this point we assume that the spherical particles are polydisperse and in this case:

$$F(Q,\overline{R_c})^2 = \int_0^\infty G(R_c)F(Q,R_c)^2 d\,R_c \quad (12)$$

Where $G(R_c)$ is the normalized probability of finding a particle with a core radius between R_c and $R_c + dR_c$,and $\overline{R_c}$ is the mean particle radius. We choose the Schulz distribution because of its mathematical simplicity and the normalized form is [6–8]

$$G(R_c) = \frac{R^Z_C}{\Gamma(Z+1)}\left(\frac{Z+1}{\overline{R_c}}\right)^{Z+1} exp\left[-\frac{R_c}{\overline{R_c}}(Z+1)\right] \quad (13)$$

Where Z is related to the polydispersity σ_c of the particle radius distribution by the expression

$$\sigma^2_c = \frac{1}{Z+1} \quad (14)$$

The size-averaged scattering function for a Schulz distributed system hard spheres follows as

$$I(Q) = \phi_c \Delta\rho^2 \left[\frac{4\pi}{(QR_c)}\right]^2 \left[\frac{1}{2} + \frac{1}{2}\frac{(Z+2)}{(Z+1)}(QR_c)^2 - \frac{1}{2}[B(2QR_c)]^{\frac{Z+1}{2}} cos[(Z+1)D(2QR_c)] - (QR_c)[B(2QR_c)]^{\frac{Z+2}{2}} sin[(Z+2)D(2QR_c)] + \frac{1}{2}(QR_c)^2 \frac{(Z+2)}{(Z+1)}[B(2QR_c)]^{\frac{Z+3}{2}} cos[(Z+2)D(2QR_c)]\right] S(Q, R_d) \quad (15)$$

Where the functions $B(2QR_c)$ and $D(2QR_c)$ are defined as:

$$B(2QR_c) = \frac{(Z+1)^2}{(Z+1)^2 + (2QR_c)^2} \quad (16)$$

$$D(2QR_c) = tan^{-1}\left(\frac{2QR_c}{Z+1}\right) \quad (17)$$

This result is for a system of non-interacting polydisperse spheres. When the particle concentration is finite, the scattering intensity also contains contributions from the inter-particle structure factor $S(Q, R_d)$ that has the simplest analytical form through the Percus-Yevick approximation for spheres interacting through a hard sphere potential. Within the Percus-Yevick approximation, $S(Q, R_d)$ is expressed as:

$$S(Q, R_d) = \frac{1}{1 - \bar{N}C(Q)} \quad (18)$$

$\bar{N}C(Q)$ is the Fourier transform of the direct correlation function given by:

$$\bar{N}C(Q) = -24\phi\left\{\lambda_1\left[\frac{\sin(2QR_d) - (2QR_d)cos(2QR_d)}{(2QR_d)^3}\right] - 6\phi\lambda_2\left[\frac{(2QR_d)^2\cos(2QR_d) - 2(2QR_d)sin(2QR_d) - 2cos(2QR_d) + 2}{(2QR_d)^4}\right] - \phi\frac{\lambda_2}{2}\left[\frac{(2QR_d)^4\cos(2QR_d) - 4(2QR_d)^3 sin(2QR_d) - 12(2QR_d)^2 cos(2QR_d) + 24(2QR_d)sin(2QR_d) + 24cos(2QR_d) - 24}{(2QR_d)^6}\right]\right\} \quad (19)$$

The parameters λ_1 and λ_2 are given by:

$$\lambda_1 = \frac{(1+2\phi)^2}{(1-\phi)^4} \quad (20)$$

$$\lambda_2 = \frac{-(1+\phi/2)^2}{(1-\phi)^4} \quad (21)$$

The detailed description of the model can be also found in [9–11].

References

[1] Krutyeva, M.; Brás, A.R.; Antonius, W.; Hövelmann, C.H.; Poulos, A.S.; Allgaier, J.; Radulescu, A.; Lindner, P.; Pyckhout-Hintzen, W.; Wischnewski, A.; et al. Association Behavior, Diffusion, and Viscosity of End-Functionalized Supramolecular Poly(ethylene glycol) in the Melt State. *Macromolecules* **2015**, *48*, 8933–8946, doi:10.1021/acs.macromol.5b02060.

[2] Folmer, B.J.B.; Sijbesma, R.P.; Versteegen, R.M.; van der Rijt, J.A.J.; Meijer, E.W. Supramolecular Polymer Materials: Chain Extension of Telechelic Polymers Using a Reactive Hydrogen-Bonding Synthon. *Adv. Mater.* **2000**, *12*, 874–878, doi:10.1002/1521-4095(200006)12:12<874::AID-ADMA874>3.0.CO;2-C.

[3] Brás, A.R.; Hövelmann, C.H.; Antonius, W.; Teixeira, J.; Radulescu, A.; Allgaier, J.; Pyckhout-Hintzen, W.; Wischnewski, A.; Richter, D. Molecular approach to supramolecular polymer assembly by small angle neutron scattering. *Macromolecules* **2013**, *46*, 9446–9454, doi:10.1021/ma401714r.

[4] Vilgis, T.A.; Benmouna, M.; Benoit, H. Static Scattering from Multicomponent Polymer Systems: Theoretical Models. *Macromolecules* **1991**, *24*, 4481–4488, doi:10.1021/ma00016a001.

[5] Hammouda, B. *A Tutorial on Small-Angle Neutron Scattering From Polymers*; National Institute of Standards and Technology: Gaithersburg, 1995.

[6] Flory, J. Molecular Size Distribution in Ethylene Oxide Polymers. *J. Am. Chem. Soc.* **1940**, *62*, 1561–1565,

doi:10.1021/ja01863a066.
[7] Schatzki, T. F. Statistical computation of distribution functions of dimensions of macromolecules. *J. Polym. Sci.* **1962**, *57*, 337–356, doi:10.1002/pol.1962.1205716526.
[8] Kotlarchyk, M.; Stephens, R. B.; Huang, J. S. Study of Schultz distribution to model polydispersity of microemulsion droplets*J. Phys. Chem.* **1988**, *92*, 1533–1538, doi:10.1021/j100317a032.
[9] Hammouda, B. *Probing Nanoscale Structures – The SANS Toolbox*; National Institute of Standards and Technology Center: Gaithersburg, 2016;

[10] Hammouda, B.; Ho, D.L.; Kline, S. Insight into clustering in poly(ethylene oxide) solutions. *Macromolecules* **2004**, *37*, 6932–6937, doi:10.1021/ma049623d.
[11] Bartlett, P.; Ottewill, R.H. A neutron scattering study of the structure of a bimodal colloidal crystal. *J. Chem. Phys.* **1992**, *96*, 3306–3318, doi:10.1063/1.461926.
